# Changes in Leaf-Level Nitrogen Partitioning and Mesophyll Conductance Deliver Increased Photosynthesis for *Lolium perenne* Leaves Engineered to Accumulate Lipid Carbon Sinks

**DOI:** 10.3389/fpls.2021.641822

**Published:** 2021-03-09

**Authors:** Luke J. Cooney, Zac Beechey-Gradwell, Somrutai Winichayakul, Kim A. Richardson, Tracey Crowther, Philip Anderson, Richard W. Scott, Gregory Bryan, Nicholas J. Roberts

**Affiliations:** Plant Biotechnology Team, AgResearch Ltd, Palmerston North, New Zealand

**Keywords:** cysteine oleosin, diacylglycerol acyl-transferase, lipid, *Lolium perenne*, photosynthesis, sink strength

## Abstract

Diacylglycerol acyl-transferase (DGAT) and cysteine oleosin (CO) expression confers a novel carbon sink (of encapsulated lipid droplets) in leaves of *Lolium perenne* and has been shown to increase photosynthesis and biomass. However, the physiological mechanism by which DGAT + CO increases photosynthesis remains unresolved. To evaluate the relationship between sink strength and photosynthesis, we examined fatty acids (FA), water-soluble carbohydrates (WSC), gas exchange parameters and leaf nitrogen for multiple DGAT + CO lines varying in transgene accumulation. To identify the physiological traits which deliver increased photosynthesis, we assessed two important determinants of photosynthetic efficiency, CO_2_ conductance from atmosphere to chloroplast, and nitrogen partitioning between different photosynthetic and non-photosynthetic pools. We found that DGAT + CO accumulation increased FA at the expense of WSC in leaves of *L. perenne* and for those lines with a significant reduction in WSC, we also observed an increase in photosynthesis and photosynthetic nitrogen use efficiency. DGAT + CO *L. perenne* displayed no change in rubisco content or V_cmax_ but did exhibit a significant increase in specific leaf area (SLA), stomatal and mesophyll conductance, and leaf nitrogen allocated to photosynthetic electron transport. Collectively, we showed that increased carbon demand *via* DGAT+CO lipid sink accumulation can induce leaf-level changes in *L. perenne* which deliver increased rates of photosynthesis and growth. Carbon sinks engineered within photosynthetic cells provide a promising new strategy for increasing photosynthesis and crop productivity.

## Introduction

Global food security remains one of the most pressing issues of our time. With increasing population and food production needs, enhancing photosynthesis represents a major target for improving crop productivity (Evans and Lawson, 2020). To address this, several bioengineering strategies have targeted improvements in the efficiency of photosynthetic energy conversion and photo-assimilate production, e.g., carbon concentrating mechanisms (Atkinson et al., 2016), C4 rice (Ermakova et al., 2020) and photorespiratory bypasses (Xin et al., 2015). While promising, the importance of photo-assimilate *utilization* to maintain photosynthetic capacity is also becoming increasingly apparent (Ainsworth and Bush, 2011; Jansson et al., 2018; Dingkuhn et al., 2020). Photosynthetic capacity is regulated by (among other things) the demand for carbon (sink strength; Paul and Foyer, 2001), with a downregulation of photosynthesis commonly reported for plants under diminished sink capacity, or under conditions of elevated carbon availability (Ainsworth et al., 2004; Bernacchi et al., 2005; Guo et al., 2006; Ribeiro et al., 2017; Ruiz-Vera et al., 2017). Carbon sinks are typically associated with reproductive or heterotrophic organs, or new growth in plants (Demmig-Adams et al., 2017). However, for agronomic production mature leaves could also offer some utility as carbon sinks, given the proximity to sites of carbon assimilation and ease of harvesting (relative to underground tissues). Increasing the sink capacity of leaves therefore represents a promising strategy to maximize the photosynthetic potential of crops.

Oils are the most energy-dense metabolites found in plants and an important carbon storage compound in seeds. By contrast, plant oils and their component fatty acids (FA) are found in low concentrations in vegetative tissues, present as signaling molecules or structural components of cell membranes (Xu and Shanklin, 2016). Elevation and stabilization of oils in leaves, as occurs in seeds, could therefore increase the sink capacity of these organs. Fortunately, several bioengineering strategies have already been developed to increase the oil content in leaves (Carlsson et al., 2011; Ohlrogge and Chapman, 2011; Dyer et al., 2012; Winichayakul et al., 2013; Vanhercke et al., 2017, 2019a). Although originally designed to increase oil yields, these technologies may also provide a useful tool to examine the relationship between additional leaf sink capacity and photosynthesis (Paul and Eastmond, 2020).

The main compound in plant oil, triacylglycerol (TAG) and FA, represent the primary targets for oil increasing bioengineering strategies. However, of the numerous and varied gene combinations reported in the literature (reviewed in Vanhercke et al., 2014, 2019a), few described changes in photosynthesis and the reason are not immediately clear. It may simply be that few groups sufficiently examined this trait, especially under conditions which deliver high carbon availability (Fan et al., 2019). Alternatively, it may be that some technologies impose too small a sink to affect photosynthesis, especially if oils are not protected and are rapidly degraded within the leaf. Conversely, too great a sink could create excessive competition for carbon, which could hinder plant development (Mitchell et al., 2020). Finally, the use of global transcription factors to enhance oil accumulation may have unintended pleiotropic effects and could impede normal cellular function (Grimberg et al., 2015). Indeed, several gene combinations reported in the literature also coincided with a growth penalty (Fan et al., 2013, 2014, 2015; Kelly et al., 2013; Yurchenko et al., 2017; Zhai et al., 2017; Yu et al., 2018; Vanhercke et al., 2019a). One notable exception is the combination of diacylglycerol acyltransferase (DGAT) and cysteine-oleosin (CO) expression which collectively increased TAG assembly and prevented lipid droplet degradation, and coincided with an increase in both photosynthesis and shoot biomass in *Arabidopsis* (Winichayakul et al., 2013). In *Lolium perenne* (perennial ryegrass), DGAT + CO expression increased FA at the expense of leaf carbohydrates, which coincided with enhanced net carbon capture and growth, especially under high N supply and elevated atmospheric CO_2_ (i.e., high carbon availability; Beechey-Gradwell et al., 2020). DGAT + CO *L. perenne* displayed a greater SLA (leaf area per unit dry weight) than control plants, a trait typically associated with low carbon availability (e.g., low light; Poorter et al., 2009; or frequent defoliation; Lee et al., 2010), and also reported for high-oil transgenic *Nicotiana tabacum* during vegetative growth (Mitchell et al., 2020). Increased SLA can provide more leaf area for light interception and gas exchange and partially explains the increased growth rate for DGAT + CO *L. perenne* (Beechey-Gradwell et al., 2020). Moreover, DGAT + CO *L. perenne* exhibited greater net photosynthesis per unit leaf area (A_area_), providing the first example of an engineered lipid carbon sink delivering increased photosynthesis in a commercially significant crop (Paul and Eastmond, 2020). Despite the potential to simultaneously increase leaf energy density (Winichayakul et al., 2020) and yield, the leaf-level physiological mechanism by which DGAT + CO increases A_area_ remains unknown.

The photosynthetic apparatus accounts for most (60–80%) of crop leaf nitrogen (N), so A_area_ often correlates with leaf N on an area basis (N_area_; Evans, 1989). Although leaf N is untested for DGAT + CO *L. perenne*, the ratio of A_area_ to N_area_, termed photosynthetic nitrogen use efficiency (PNUE) is typically higher for high SLA species, achieved *via* (among other factors) greater internal conductance to CO_2_ (g_m_) and within-leaf N allocation to rate-limited photosynthetic functions (Poorter and Evans, 1998; Hikosaka, 2004; Onoda et al., 2017). Given the increased SLA reported for DGAT + CO *L. perenne* (Beechey-Gradwell et al., 2020), changes to PNUE, g_m_, and within-leaf N allocation may similarly explain the reported increase in A_area_. To examine the relationships between DGAT + CO leaf lipid sinks, leaf N, and photosynthesis, we carried out three experiments to test three specific hypotheses; (Hypothesis i) that increased photosynthesis for DGAT + CO will depend upon the level of DGAT + CO accumulation (i.e., sink strength; experiment 1); (Hypothesis ii) that DGAT + CO accumulation increases PNUE (experiments 1 and 2); (Hypothesis iii) that DGAT + CO accumulation increases g_m_ and within-leaf N allocation to photosynthetic pools (experiment 3). In experiment 1, we compared leaf FA, water-soluble carbohydrates (WSC), leaf N, relative growth rates, and photosynthesis in multiple DGAT + CO *L. perenne* lines, varying in DGAT + CO accumulation. In experiment 2, we examined leaf nitrogen and photosynthesis for DGAT + CO *L. perenne* grown under five levels of external nitrogen supply (1–7.5 mM NO_3_^−^). Finally, in experiment 3, we compared rubisco content, mesophyll conductance and the proportions of N allocated between photosynthetic and non-photosynthetic pools for DGAT + CO *L. perenne* and non-transformed (NT) controls.

## Materials and Methods

### Plant Transformation

The coding sequences for CO and DGAT (S205A mutation; Winichayakul et al., 2013) were optimized for expression in rice and placed in a back-to-back orientation under the control of the rice CAB and RUBISCO small subunit promoters, respectively. For *Agrobacterium*-mediated transformation, the expression cassette was cloned into the pCAMBIA1300 binary vector, while for particle bombardment, the cassette was cloned into a pUC-based vector.

Transformed lines were generated from *L. perenne* callus induced from immature inflorescences and transformed by Agrobacterium-mediated transformation (DGAT + CO1-4) or particle bombardment (DGAT + CO5). Plants from *Agrobacterium*-mediated transformation were generated as per Bajaj et al. (2006) while plants from microprojectile bombardment were generated as per Altpeter et al. (2000).

### Experiment 1 Design and RGR Analysis

In experiment 1, we examined multiple DGAT + CO lines, varying in DGAT + CO accumulation. Five DGAT + CO ryegrass lines were selected from three genetic backgrounds (i.e., two DGAT + CO lines were generated from an “Alto” cultivar individual and three DGAT + CO lines were generated from two “Impact” cultivar individuals) and propagated asexually *via* the production of clonal ramets as per Beechey-Gradwell et al. (2020). Each DGAT + CO line was then designated an arbitrary label, DGAT + CO1-5 [DGAT + CO5 was previously reported as either “HL” or “6205” by Beechey-Gradwell et al. (2020)]. To eliminate growth form or tiller age differences between ramets, all DGAT + CO lines, and respective non-transformed (NT) controls, underwent three rounds of propagation. During each round, five ramets of five tillers each were potted and grown for 4 weeks. All plants were grown in a controlled temperature room with ~600 μmol photons m^−2^ s^−1^ red/blue light provided by 600W NanaPro LED lights (LEDgrowlights, Hamilton, New Zealand), 20°C/15°C day/night temperature and 12 h photoperiod, with humidity uncontrolled and commonly fluctuating between 65 and 75% during the day and 80 and 90% at night. In Jan 2019, 40 × 5-tiller ramets were produced for each line, 10 of which were immediately harvested to confirm comparable starting weights (Supplementary Table S1). The remaining 30 ramets per line were transplanted into 1.3 L sand and grown for 3 weeks to establish a root system. During this “establishment phase,” pots were flushed thrice weekly with 100 ml of basal nutrient media described in Andrews et al. (1989) containing N as 2 mM KNO_3_. Following the establishment phase, shoot material was harvested 5 cm above the sand and used to rank plants from smallest to largest. The five smallest and five largest plants per line were discarded and 10 of the remaining 20 plants per line were randomly selected and the remaining shoots (0–5 cm above pot surface) and roots were harvested, oven dried and weighed (post-establishment harvest). The remaining 10 plants per line were grown for another 3 weeks, with 4 mM NH_4_NO_3_ applied as described above, and harvested (final harvest). Relative growth rate was calculated as per Poorter (1989); RGR = (ln W_2_–ln W_1_)/(t_2_–t_1_) where W_1_ = post-establishment dry weight, W_2_ = final harvest dry weight, t_1_ = day 22 and t_2_ = day 43.

### Experiments 2 and 3 Design

Experiments 2 and 3 provide a detailed analysis of a single transgenic high lipid line, DGAT + CO5, and the corresponding control, NT3. Experiment 2 relates to unreported leaf N and gas exchange data from plants grown at ambient CO_2_ as part of a larger experiment described in Beechey-Gradwell et al. (2020). Plant growth conditions, preparation and establishment for experiment 2 was similar to experiment 1 [additional details provided in Beechey-Gradwell et al. (2020)], however, during the regrowth phase, different N treatments were introduced, and plants were regrown under one of five levels of NO_3_^−^ (1–7.5 mM).

For experiment 3, in which we examined rubisco contents, mesophyll conductance and within-leaf N allocation (details below), an additional 12 ramets of DGAT + CO5 and NT3 were prepared and grown as per experiment 1 with two minor alterations; regrowth phase N supply was delivered as 5 mM NO_3_^−^, and growth irradiance, provided by the above LEDs, had reduced slightly to ~550 μmol photons m^−2^ s^−1^.

### SDS-PAGE Immunoblot Analysis of DGAT + CO (Experiment 1)

Protein samples were prepared by collecting four fresh *L. perenne* leaf blades (approximately 2 cm long) in a 2-ml screw cap micro tube containing 150 μl of sterile H_2_O, 200 μl of 2x protein loading buffer [1:2 diluted 4x lithium dodecyl sulphate (LDS) sample buffer (Life Technologies, Carlsbad, CA, United States)], 8 M urea, 5% (v/v) β-mercaptoethanol, and 0.2 M dithiothreitol and 40 μl of NUPAGE^™^ sample reducing agent (NP0009, ThermoFisher Scientific, Waltham, MA, United States). The mixtures were homogenized using the Bead Ruptor 24 model (Omni International, Kennesaw, GA, United States). The samples were heated at 70°C for 10 min, centrifuged at 20,000 *g* for 30 s and collected for the soluble protein suspension. Equal quantities of proteins were determined and separated by SDS-PAGE (Mini-PROTEAN® TGX stain-free^™^ precast gels; Bio-Rad, Hercules, CA, United States) and blotted onto Bio-Rad polyvinylidene difluoride (PVDF) membrane for the DGAT1-V5 immunoblotting. Equivalent amounts of proteins were separated on gradient 4–12% Bis-Tris gel (NUPAGE; Life Technologies) and blotted onto nitrocellulose membrane for the CO immunoblotting. Immunoblotting was performed as described previously in Winichayakul et al. (2013). Chemiluminescent activity was developed using WesternBright ECL spray (Advansta, Menlo Park, CA, United States) and visualized by ChemiDoc^™^ imaging system (Bio-Rad Laboratories Inc.). Volume intensity of monomeric forms of the protein was quantified using Image Lab^™^ software for PC version 5.2.1 (Bio-Rad Laboratories Inc.).

### Photosynthetic Gas Exchange (Experiments 1–3)

Gas exchange measurements for each experiment were completed 2–3 weeks after the post-establishment defoliation. Three tillers were selected per plant and the youngest fully expanded leaves (determined by the appearance of a leaf collar and selected to minimize the effects of self-shading) of each were simultaneously acclimated in the leaf chamber of either a LI-COR 6800 (experiments 1 and 3) or a LI-COR 6400 infrared gas exchange system (LI-COR Biosciences Ltd., Nebraska, United States; experiment 2) under the following conditions; 400 ppm CO_2_, 70% relative humidity, 20°C and PAR of either 600, 1500 or 550 μmol photons m^−2^ s^−1^ red/blue light (for experiments 1, 2, and 3, respectively). After 20 min, net photosynthesis (A_area_), stomatal conductance (g_s_) and transpiration (E) were measured.

For experiment 3, mesophyll conductance (g_m_) was calculated *via* the variable J method (Harley et al., 1992) using

$$g_{m}=\frac{A}{C_{i}-\frac{\Gamma *\left[J+8\;\left(A+R_{d}\right)\right]}{J-4\;\left(A+R_{d}\right)}}\;$$ (1)

where J was derived from chlorophyll fluorescence (quantum efficiency of PSII × PAR × PSII absorbance). PSII absorbance was assumed to be half of leaf absorbance (Pons et al., 2009), measured as Chl_A+B_/(Chl_A+B_ + 76) as in Evans and Poorter (2001) where Chl_A+B_ is total chlorophyll per unit leaf area (the quantification of which is described below). *J* was then adjusted according to Pons et al. (2009) using the relationship between *J* derived *via* chlorophyll fluorescence and *J* derived *via* gas exchange (4 x gross assimilation) under non-photorespitory conditions. Accordingly, A-C_i_ curves were performed under photorespitory and non-photorespitory conditions as follows; ambient O_2_ A-C_i_ curves were performed first using the chamber conditions described above and the following CO_2_ concentrations; 400, 300, 200, 100, 50, 0, 400, 400, 400, 600, 700, 800, 900, 1000, and 1200. At each step, leaves were given 3 min to acclimate before data logging. The air supply was then immediately switched to 2% O_2_ provided by supplementary gas (2% O_2_ in N; BOC Limited, NSW, Australia) *via* the main console air inlet with a flow meter used to confirm positive air flow. Leaves were given an additional 30 min to acclimate to low O_2_ before the A-C_i_ procedure was repeated. Rapid light response curves were used for determination of R_d_ *via* the Kok method (Kok, 1948), modified after Yin et al. (2011); the same ambient O_2_ chamber conditions described above were used except leaves were acclimated under saturating PAR, 1500 μmol photons m^−2^ s^−1^, before dropping PAR to 0 across 10 steps with 5 min acclimation at each. R_d_ was then substituted into the regression equation of the initial A-C_i_ curve to solve for C_i_*(Brooks and Farquhar, 1985). Γ* was solved simultaneously with g_m_ by substituting Γ* with C_i_* + R_d_/g_m_ into equation 1 (Warren, 2006). This delivered a single converging value for each g_m_ and Γ*. V_cmax_ was derived using the A-C_i_ analysis excel tool (Sharkey, 2016) with rate-limitation assigned using chlorophyll fluorescence (Sharkey, 2016; Supplementary Figure S1), and *g*_m_ and *R*_d_ fixed as determined above. Slow light-response curves were completed for determination of *J*_max_ (Sharkey, 2016). Again, the chamber conditions described above were used, however PAR increased from 0 to 1500 μmol photons m^−2^ s^−1^ across 10 steps with 30 min acclimation at each. *J*_max_ was then derived from the light response analysis of the Sharkey excel tool (Sharkey, 2016), with *g*_m_ and *R*_d_ fixed.

For all gas exchange measurements, bulk flow leaks were periodically checked by blowing on the leaf chamber. Diffusion leaks through the chamber gaskets were minimized by performing all low CO2 measurements with the Licor 6800, which displays a lower leak rate coefficient than the 6400XT. After all gas exchange analyses, leaves were then removed and photographed. Leaf area was calculated using GIMP 2.8.22 (GNU Image Manipulation Program^1^). Leaves were then dried and weighed, and specific leaf area calculated as SLA = LA/DW and photosynthesis per unit leaf mass calculated as A_mass_ = SLA × A.

### Fatty Acid Analysis (Experiment 1)

Leaf material was collected on the final day of the experiment, freeze dried and ground *via* bead mill. Ten milligram was sub-sampled per plant and from this, FA were extracted in hot methanolic HCl (modified after Browse et al., 1986). FA were quantified by GC-MS (QP 2010 SE, Shimadzu Corp., Kyoto, Japan) against an internal standard of 10 mg C15:0 and total FA were calculated as the sum of palmitic acid (16:0), palmitoleic acid (16:1), stearic acid (18:0), oleic acid (18:1), linoleic acid (18:2) and linolenic acid (18:3).

### Sugar Quantification (Experiment 1)

Total water-soluble carbohydrates (WSC) were analyzed using the anthrone method (Hedge and Hofreiter, 1962). Leaves were sampled at midday and immediately frozen in liquid nitrogen. Using 25 mg freeze-dried, ground leaf material, low molecular weight carbohydrates (LMW; including glucose, fructose, sucrose and some LMW fructans; Pollock and Jones, 1979) were twice extracted in 1 ml, 4:1 EtOH: H_2_O at 65°C for 30 min, centrifuged and supernatant collected and combined at each extraction. High molecular weight carbohydrates (HMW; this fraction includes HMW fructans, the main storage sugar in *L. perenne*; Pollock and Jones, 1979) were then twice extracted in 1 ml H_2_O at 65°C for 30 min, centrifuged and supernatant collected and combined at each extraction. The soluble carbohydrate extracts were mixed with anthrone reagent (Sigma-Aldrich, St Louis, MO, United States) for 25 min at 65°C, A_620_ determined using a Versamax tunable plate reader (Molecular Devices Corporation, Sunnyvale, CA, United States) and compared to LMW and HMW standards, prepared using sucrose and inulin, respectively.

### Leaf Nitrogen Biochemistry Including Rubisco and Chlorophyll Quantification (Experiment 3)

Total soluble protein was quantified according to Bradford (1976). Using 500 mg leaf FW ground in liquid N, soluble protein was twice extracted in 15 ml of 50 mM sodium phosphate buffer (pH 7) containing 5 mM DTT, centrifuged (4000 *g*, 15 min, 4°C) and 5 μl of the supernatant combined with 200 μl Bio-Rad protein assay dye (Bio-Rad, CA, United States). Absorbance was measured at 595 nm using bovine serum albumin (MP biomedical, Auckland, New Zealand) as the protein standard. Rubisco was determined according to Makino et al. (1986) with minor modifications. The total soluble protein extract (20 μl) was combined (1:1) with 2x Laemmli Buffer (Sigma S3401-10VL, St Louis, MO, United States) and heated at 95°C for 5 min. Protein samples were then separated by SDS-PAGE (Bio-Rad, 4–15% Mini-PROTEAN TGX Stain-free) for 30 min at 180 V and the resulting gels stained with 0.25% CBB-R dye in 40% methanol and 10% acetic acid solution overnight, and then rinsed repeatedly with 40% methanol and 10% acetic acid solution until the background was colorless. Large (52 kDa) and small (15 kDa) Rubisco subunits were excised from the gel and transferred into tubes with 0.75 ml of formamide and shaken at 50°C for 6 h. The absorbance of the formamide extracts was measured at 595 nm using the background gel as a blank and bovine serum albumin as the protein standard. N associated with soluble protein (N_S_) and rubisco (N_R_) was calculated assuming protein contains 16% N.

Using 200 mg leaf FW, ground in liquid N, chlorophylls were twice extracted in 10 ml 95% EtOH, then stored for 6–8 h in the dark with regular vortexing, and then centrifuged (4000 *g*, 15 min, 4°C). The supernatant was removed, diluted 2-fold and absorbance peaks measured using a Versamax tunable plate reader using the pathlength correction formula described in Warren (2008). Chlorophyll concentrations were determined from A_648_ and A_664_ using the formula described in Lichtenthaler (1987). N associated with pigment-protein complexes (N_P_) was calculated assuming 37.3 mol N mol^−1^ total Chl (Evans and Clarke, 2019).

Nitrogen associated with electron transport and ATP synthesis (collectively “bioenergetics”; N_E_) was calculated indirectly from electron transport capacity [J_max_; the calculation of which was described above and here adjusted to 25°C as per June et al. (2004)]. A linear correlation between cytochrome *f* (cyt *f*) content per unit leaf area and J_max_ at 25°C, of 155 mol electron mol^−1^ cyt f s^−1^ was assumed (Evans, 1987). Recently revised ratios of cyt *f* to the other components of electron transport and ATP synthesis were used to calculate an N_E_ cost of 10.86 mol N mmol^−1^ cyt *f* (Evans and Clarke, 2019).

Total N concentration (N_mass_) was determined on 200 mg of dried, ground samples using a CN elemental analyzer (Elementar VarioMax CN analyzer, Hanau, Germany). “Remaining” leaf N (N_O_) was calculated as N_mass_ – N_S_ – N_P_ – N_E_. Approximately 1 g fresh subsamples were weighed, oven-dried, and weighed again for converting the above FW measurements to DW basis, and then converted onto a leaf area basis by dividing by SLA.

### Statistical Analysis

All statistical analyses as well as normality and variance tests were performed using R version 3.3.3 (R Foundation, Vienna, Austria). For experiment 1, two-way factorial ANOVAs were used to evaluate the relationship between each of the following dependent variables: FA, WSC, gas exchange parameters, biomass, RGR, SLA and independent factors: genetic background (3 levels) and line (8 levels, i.e., 3 NT and 5 DGAT + CO). Tukey tests were used for *post-hoc* analysis. Kruskall-Wallis tests were used to evaluate the effect of DGAT + CO on non-normal variables, projected leaf area, and their values of *p* were adjusted using a Bonferroni correction. Linear regressions were used to evaluate the relationship between recombinant protein contents and FA. One-way ANOVAs were used to compare LMW and HMW carbohydrates of NT1-3 with Tukey tests used for *post-hoc* analysis. For experiment 2, a forward stepwise regression was used to evaluate the relationship between genotype (NT3 and DGAT + CO5), NO_3_^−^ supply (treated as continuous) and dependent variables: A_sat_, SLA, leaf N_mass_, N_area_, g_s_ and PNUE. Variables and interaction terms with a value of *p* < 0.05 were retained in the final model. Quadratic terms were tested in each of the models to account for non-linear responses to NO_3_^−^ supply or leaf N. The same procedure was used for investigating the relationships between genotype, WSC, and leaf N content on photosynthesis. For experiment 3, NT3 and DGAT + CO5 were compared using a Student’s *t*-test or Wilcoxon rank sum test.

## Results

### Experiment 1: Comparing Multiple DGAT + CO Lines Varying in DGAT + CO Accumulation

**Figure 1:**
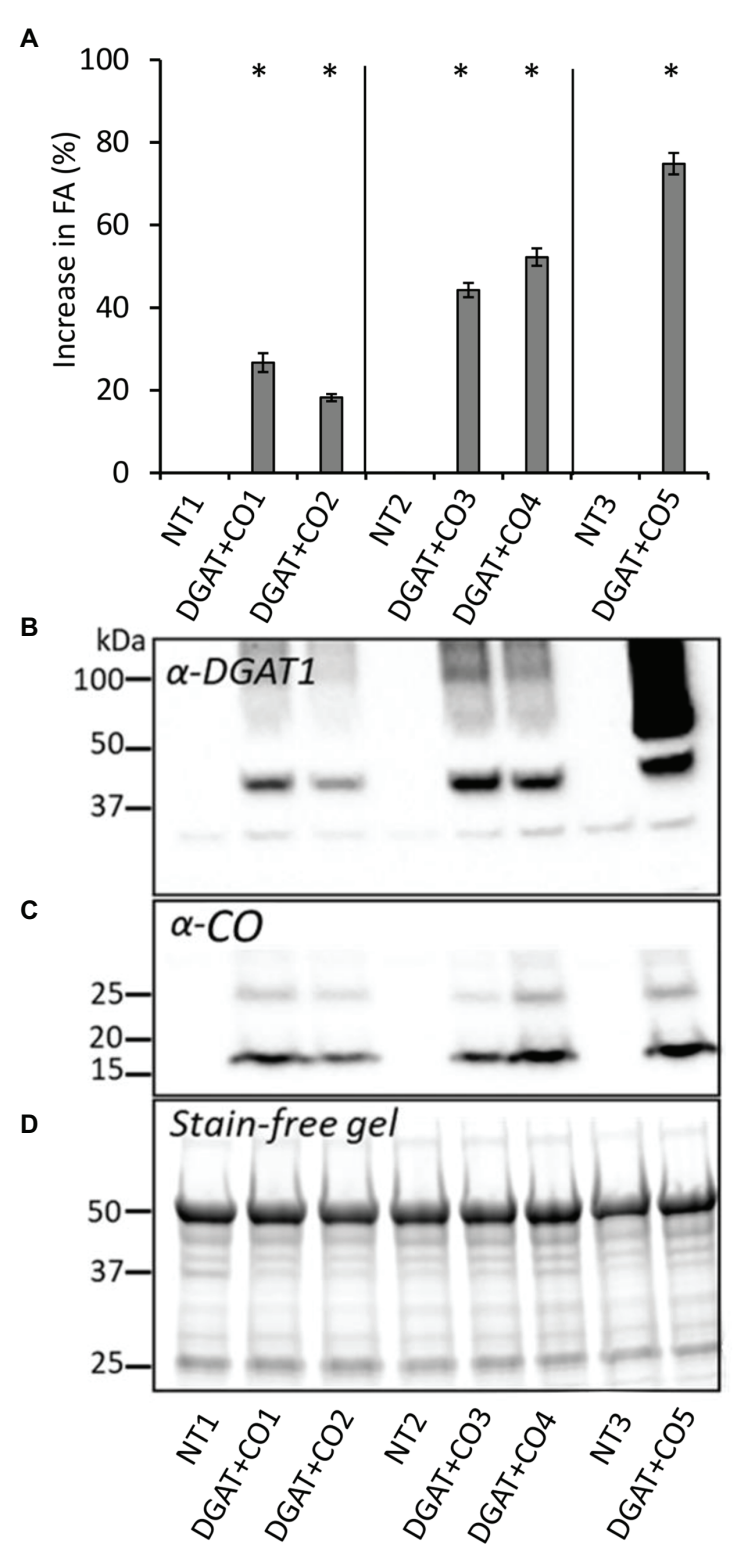
Percent difference (±SE) in leaf fatty acids compared to respective NT **(A)**, recombinant protein contents for diacylglycerol acyl-transferase (DGAT; **B**) and cysteine-oleosin **(C)**, and stain free gel showing equal protein loading for each cell **(D)**, for five DGAT + CO lines and three respective NT controls. Matching genetic backgrounds are grouped together. ^*^p < 0.05.

Five DGAT + CO lines were examined here, two (DGAT + CO1-2) transformed using an “Alto” cultivar individual (NT1), and three transformed using two “Grasslands Impact” cultivar individuals (DGAT + CO3-4 background, NT2; DGAT + CO5 background, NT3). For each DGAT + CO line, the presence of transgenic proteins was confirmed *via* SDS-page immunoblot analysis (Figure 1). All DGAT + CO lines displayed a significant increase in leaf fatty acids (FA), ranging from 118 to 174% of respective non-transformed (NT) controls (Figure 1). For the DGAT + CO lines, total leaf FA represented 4.8–5.5% of leaf dry weight (DW), whereas NT controls ranged from 2.9 to 4% of leaf DW (Table 1). The relative increase in total FA for each line, compared to respective NT control, strongly correlated with DGAT accumulation (*r*^2^ = 0.82, *p* = 0.03), but was not statistically significant for CO (*r*^2^ = 0.67, *p* > 0.05). The composition of FA was significantly altered by DGAT + CO expression, with all lines exhibiting a significant increase in C18:1 and C18:2 and a decrease in C16:0, C16:1, and C18:3 (Table 1).

**Table 1:** Fatty acid profile (% total FA) and total fatty acids (% Leaf DW) of five DGAT + CO lines and three non-transformed (NT) controls.

	C16:0	C16:1	C18:0	C18:1	C18:2	C18:3	Total FA (%DW)
NT1	11.52 (± 0.1)	2.22 (± 0.04)	1.04 (± 0.01)	1.66 (± 0.04)	12.8 (± 0.1)	70.76 (± 0.2)	4.04 (± 0.1)
DGAT + CO1	10.41 (± 0.1)^**^	1.96 (± 0.08)^**^	1.05 (± 0.02)	5.8 (± 0.2)^**^	19.95 (± 0.2)^**^	60.83 (± 0.4)^**^	5.12 (± 0.1)^**^
DGAT + CO2	10.67 (± 0.02)^**^	1.99 (± 0.04)^**^	0.91 (± 0.02)^**^	5.29 (± 0.1)^**^	18.56 (± 0.1)^**^	62.58 (± 0.2)^**^	4.78 (± 0.03)^**^
NT2	11.38 (± 0.1)	2.7 (± 0.05)	0.99 (± 0.02)	1.44 (± 0.05)	13.96 (± 0.3)	69.52 (± 0.3)	3.64 (± 0.1)
DGAT + CO3	10.38 (± 0.1)^**^	2.47 (± 0.05)^**^	0.96 (± 0.03)	4.28 (± 0.05)^**^	18.7 (± 0.1)^**^	63.2 (± 0.1)^**^	5.25 (± 0.06)^**^
DGAT + CO4	8.95 (± 1)^**^	2.07 (± 0.2)^**^	0.88 (± 0.1)	6.36 (± 0.2)^**^	22.49 (± 0.1)^**^	57.97 (± 0.2)^**^	5.54 (± 0.08)^**^
NT3	13.03 (± 0.1)	2.2 (± 0.05)	0.93 (± 0.01)	1.05 (± 0.02)	14.62 (± 0.2)	68.17 (± 0.2)	2.92 (± 0.1)
DGAT + CO5	12.29 (± 0.1)^**^	2.01 (± 0.04)^**^	0.97 (± 0.02)	3.63 (± 0.1)^**^	22.47 (± 0.1)^**^	58.61 (± 0.2)^**^	5.11 (± 0.1)^**^

Means ± SE.

** indicates statistically significant difference from respective NT control (*p* < 0.01). *n* = 10. Lines with matching genetic backgrounds are grouped together.

**Figure 2:**
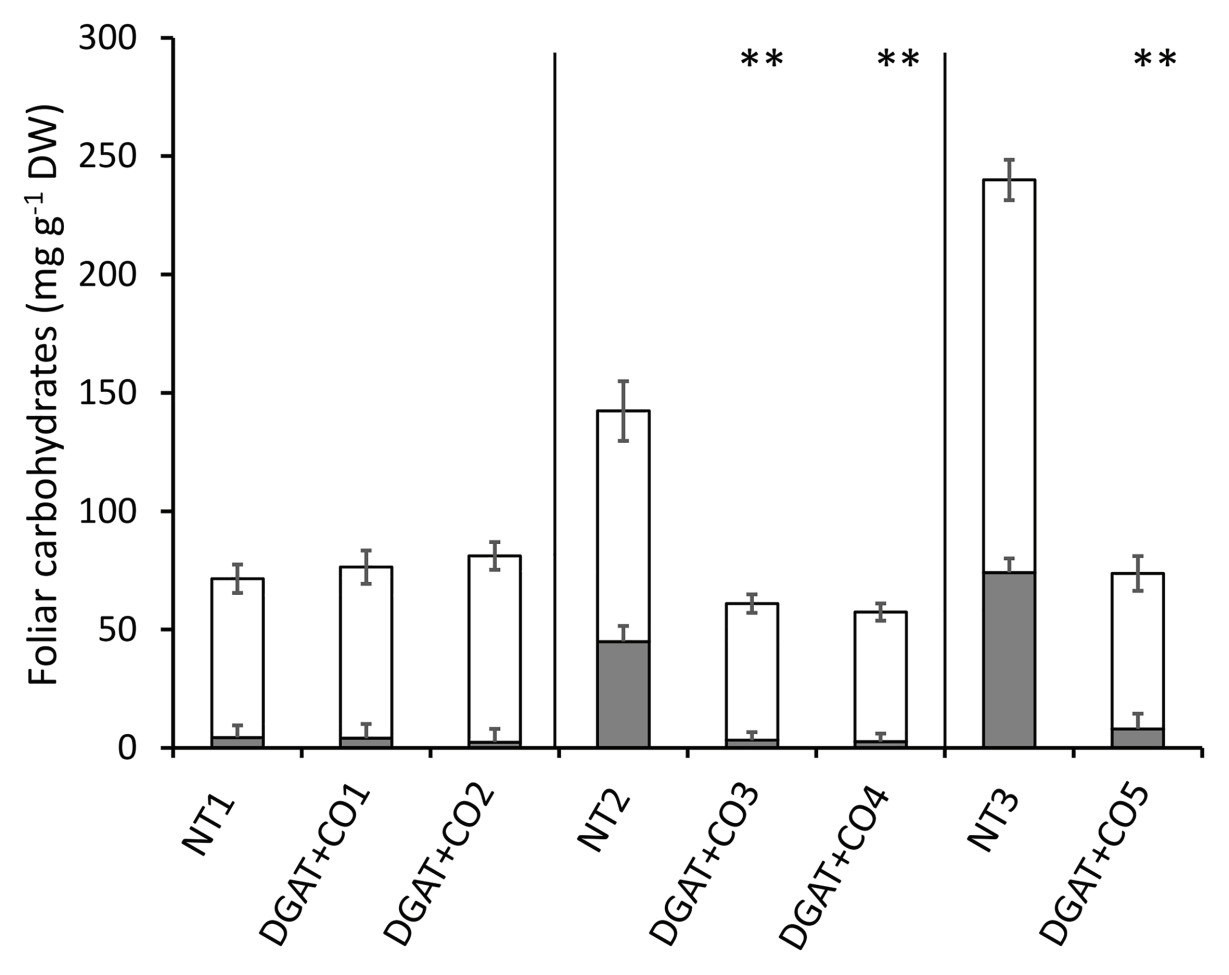
Stacked means (±SE) of high molecular weight carbohydrates (shaded grey, 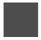) and low molecular weight carbohydrates (shaded white, 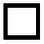) in the leaves of five DGAT + CO transformed *L. perenne* lines and respective non-transformed controls. Matching genetic backgrounds are grouped together. *n* = 10. **Statistically differs from NT, *p* < 0.01.

Leaf low molecular weight carbohydrates and HMW were significantly lower in DGAT + CO3-5, compared to respective NT controls (Figure 2), resulting in a reduction in total leaf WSC of 57–69% (Figure 2). In contrast, there were no statistical differences in LMW, HMW, or total WSC between DGAT + CO1, DGAT + CO2, and the NT1 control (Figure 2). Both LMW and HMW carbohydrates were significantly lower for NT1 compared to both NT2 and NT3 and for NT2 compared to NT3 (*p* < 0.01).

**Figure 3:**
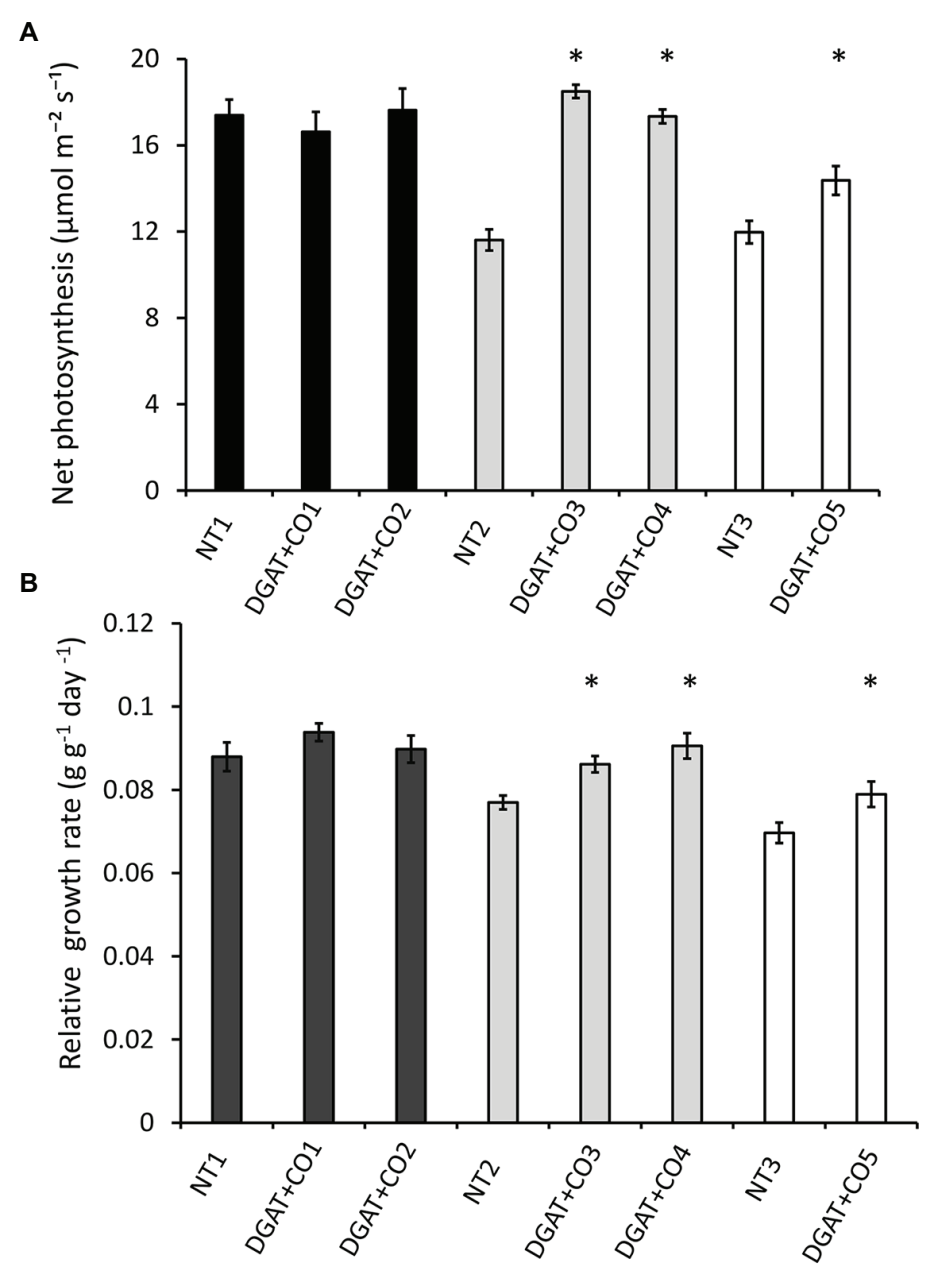
Net photosynthesis **(A)** and whole-plant relative growth rate **(B)** for five DGAT + CO lines and three NT lines. Means ± SE. ^*^Statistically differs from NT, *p* < 0.05; *n* = 10. Matching genetic backgrounds are shaded together.

Of the five DGAT + CO lines examined here, two (DGAT + CO1 and DGAT + CO2) showed no significant difference in gas exchange or biomass, compared to their respective NT control (Figure 3; Table 2) In contrast, DGAT + CO3-5 were between 59 and 82% larger than their respective NT controls at the final harvest, displaying a significant increase in shoot, root and total plant DW (Table 2). Differences in establishment growth (i.e., growth in the 3 weeks following propagation) explained some of the total growth difference for these lines (Supplementary Table S1); however, the relative growth rate (RGR) between the post-establishment harvest (3 weeks after propagation) and final harvest (6 weeks after propagation) was also significantly greater for DGAT + CO3-5, compared to respective controls (Figure 3). SLA was significantly greater for DGAT + CO5 compared to NT3, but not for DGAT + CO1-4 (Table 2). Regardless, DGAT + CO3-5 all displayed a significant increase in projected total leaf area (leaf DW × SLA), compared to respective NT controls (Table 2).

**Table 2:** Growth and gas exchange parameters for five DGAT + CO lines and three NT control lines 3 weeks after defoliation.

	Leaf DW (g)	Root DW (g)	Shoot DW (g)	Total DW (g)	LA (cm^2^)	SLA (cm^2^ g^−1^)	g_S_ (mol m^−2^ s^−1^)	E (mol m^−2^ s^−1^)
NT1	1.6 (± 0.1)	0.9 (± 0.06)	2.8 (± 0.1)	3.7 (± 0.1)	444 (± 19)	274 (± 8)	0.27 (± 0.01)	2.1 (± 0.1)
DGAT + CO1	1.6 (± 0.04)	0.7 (± 0.03) *	2.7 (± 0.1)	3.4 (± 0.1)	451 (± 15)	283 (± 9)	0.26 (± 0.02)	2 (± 0.2)
DGAT + CO2	1.6 (± 0.05)	0.9 (± 0.07)	2.7 (± 0.1)	3.6 (± 0.2)	454 (± 11)	284 (± 8)	0.28 (± 0.02)	2.1 (± 0.2)
NT2	0.8 (± 0.03)	0.4 (± 0.03)	1.4 (± 0.1)	1.9 (± 0.1)	206 (± 15)	260 (± 15)	0.15 (± 0.01)	1.2 (± 0.1)
DGAT + CO3	1.2 (± 0.03)^**^	0.9 (± 0.05)^**^	2.4 (± 0.1)^**^	3.3 (± 0.1)^**^	359 (± 9)^**^	290 (± 5)	0.34 (± 0.01)^**^	2.5 (± 0.1)^**^
DGAT + CO4	1.4 (± 0.1)^**^	0.9 (± 0.06)^**^	2.5 (± 0.1)^**^	3.4 (± 0.1)^**^	415 (± 19)^**^	287 (± 8)	0.3 (± 0.01)^**^	2.2 (± 0.1)^**^
NT3	0.9 (± 0.1)	0.5 (± 0.04)	2.1 (± 0.1)	2.5 (± 0.2)	197 (± 17)	213 (± 8)	0.0.18 (± 0.02)	1.5 (± 0.2)
DGAT + CO5	1.3 (± 0.1)^*^	0.8 (± 0.06)^**^	3.2 (± 0.2)^**^	4 (± 0.3)^**^	433 (± 31)^**^	343 (± 9)^**^	0.32 (± 0.03)^**^	2.6 (± 0.2)^**^

LA, leaf area; SLA, specific leaf area; g_s_, stomatal conductance; E, transpiration. Means ± SE. * or ** indicates statistically significant difference from respective non-transformed control (*p* < 0.05 and 0.01 respectively). *n* = 10. Lines with matching genetic backgrounds are grouped together.

**Figure 4:**
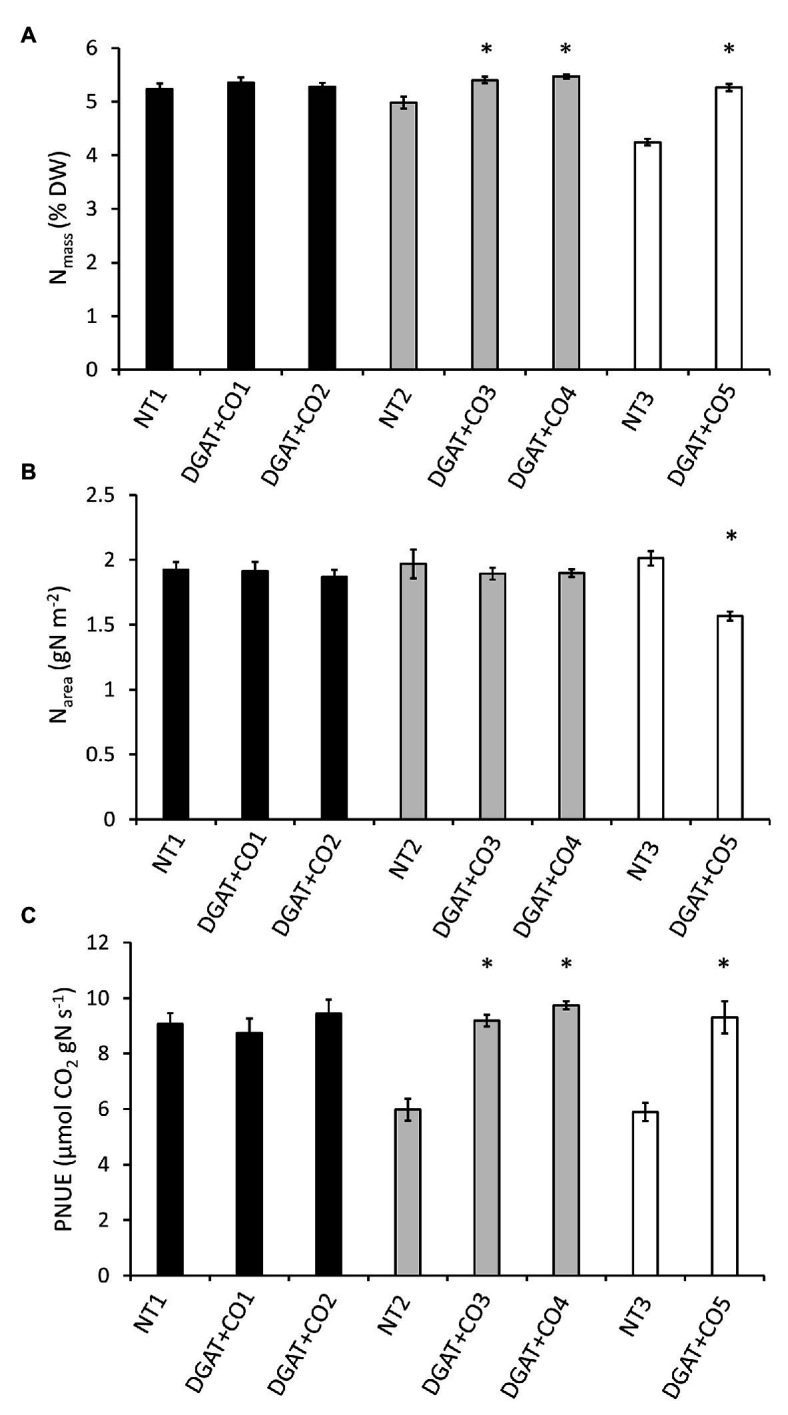
Leaf N concentration (N_mass_; **A**), N per unit leaf area (N_area_; **B**) and photosynthetic nitrogen use efficiency measured at 600 μmol photons m^−2^ s^−1^ (PNUE_amb_; **C**) for five independently transformed DGAT + CO *L. perenne* lines and respective NT controls. Means ± SE. ^*^Statistically differs from NT, *p* < 0.05; *n* = 10. Matching genetic backgrounds are shaded together.

**Figure 5:**
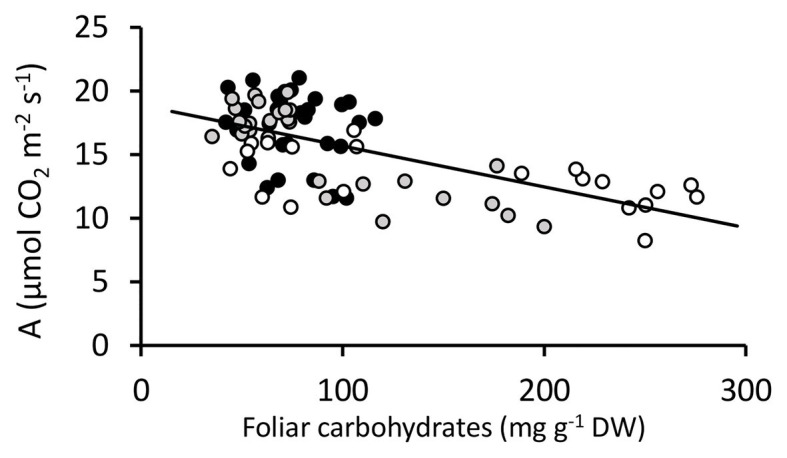
Photosynthesis vs. foliar carbohydrates for DGAT + CO and NT *Lolium perenne*. Lines from each genetic background are shaded together irrespective of DGAT + CO or NT; NT1 and DGAT + CO1-2 (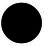), NT2 and DGAT + CO3-4 (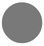) and NT3 and DGAT + CO5 (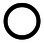). Trendline represents NT2 and NT3 derived lines. Photosynthesis measured at 600 μmol photons m^-2^ s^-1^.

DGAT + CO3-5 all displayed a significant increase in net photosynthesis (A_area_; Figure 3), transpiration (E) and stomatal conductance (g_s_; Table 2), compared to respective NT controls. Similarly, DGAT + CO3-5 exhibited significantly greater leaf N_mass_ than controls (Figure 4), whereas no difference in gas exchange or N_mass_ was identified between DGAT + CO1-2 and NT1 (Figure 4). When N was expressed on a leaf area basis (N_area_), there was no significant difference between DGAT + CO1-4 and respective controls, but there was a significant decrease in N_area_ for DGAT + CO5 relative to NT3 (Figure 4). A significant increase in PNUE (A_area_/N_area_) was observed for DGAT + CO3-5 (Figure 4), which was not observed for DGAT + CO1-2. Net photosynthesis correlated negatively with foliar carbohydrates for those plants derived from NT2 (NT2 and DGAT3-4; *r*^2^ = 0.3; *p* < 0.001) and NT3 (NT3 and DGAT + CO5; *r*^2^ = 0.7; *p* < 0.001), but not NT1 (NT1 and DGAT + CO1-2; *p* = 0.4; Figure 5).

### Experiment 2: DGAT + CO5 A-N Relationships Across NO_3_^−^ Supply Range

**Figure 6:**
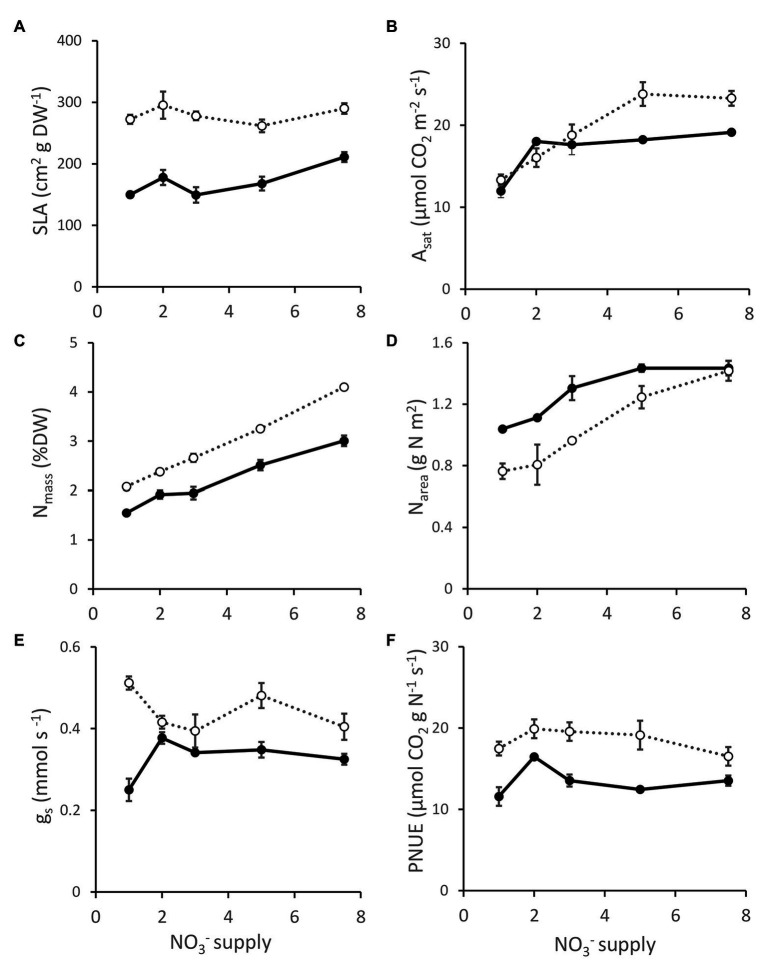
Specific leaf area (SLA; **A**), photosynthesis at 1500 μmol photons m^-2^ s^-1^ (A_sat_; **B**), leaf N concentration (N_mass_; **C**), N per unit leaf area (N_area_; **D**), stomatal conductance (g_s_; **E**) and photosynthetic nitrogen use efficiency (PNUE_sat_; **F**) for *L. perenne* DGAT + CO5 (open circles 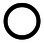) and NT control (NT3; closed circles 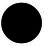) grown under 1-7 mM NO_3_^-^ supply. Means ± SE; *n* = 3 for 1, 2, and 3 mM treated plants, *n* = 5 for 5 and 7.5 mM treated plants.

DGAT + CO5 had a greater SLA than NT3 at all levels of NO_3_^−^ supply (Genotype effect, *p* < 0.001). A_sat_ was comparable between DGAT + CO5 and NT3 at 1-3 mM NO_3_^−^ supply but significantly greater for DGAT + CO5 at 5–7.5 mM NO_3_^−^ supply (Genotype x concentration interaction, *p* < 0.01; Figure 6B). Stomatal conductance (g_s_) was unaffected by NO_3_^−^ supply and was consistently greater for DGAT + CO5 than for NT3 (Genotype effect, *p* < 0.001; Figure 6E). DGAT + CO5 had a greater leaf N_mass_ than NT3 and this difference became progressively larger with increasing NO_3_^−^ supply (Genotype × concentration interaction, *p* < 0.001; Figure 6C). However, N_area_ was greater for NT3 than for DGAT + CO5 from 1 to 5 mM NO_3_^−^ supply and was similar for the two genotypes at the 7.5 mM NO_3_^−^ supply (Genotype × concentration interaction, *p* < 0.05; Figure 6D). DGAT + CO5 exhibited a greater PNUE than NT3 across the entire NO_3_^−^ supply range (Genotype effect, *p* < 0.001; Figure 6F).

**Figure 7:**
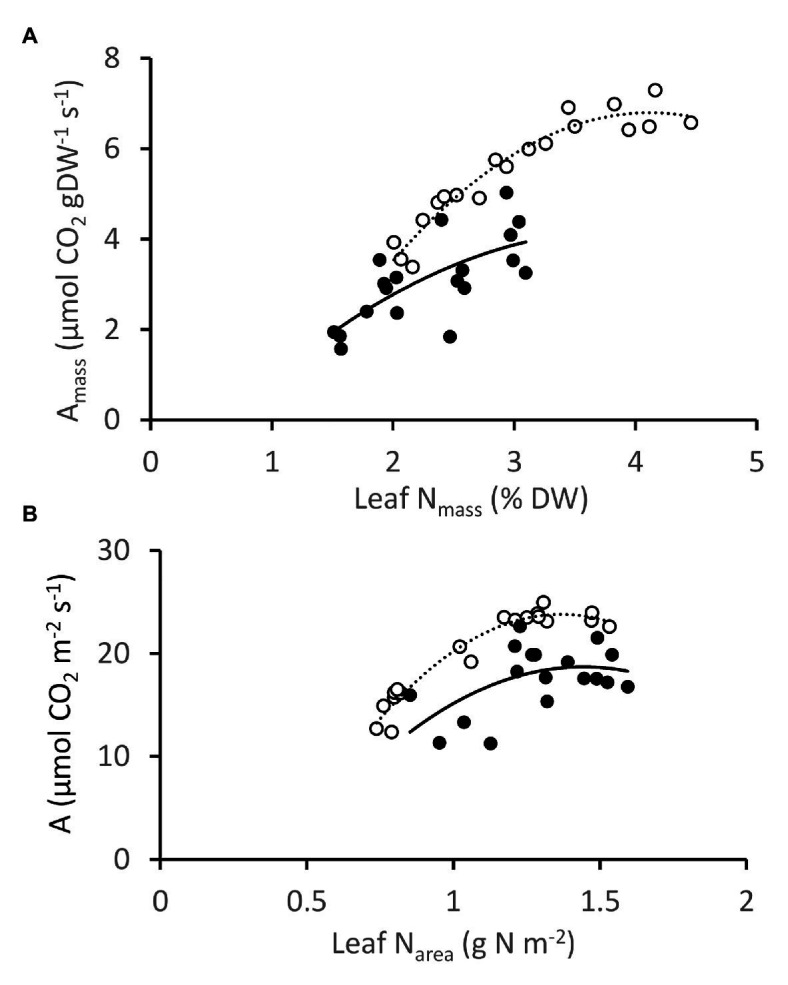
Photosynthesis vs. leaf N, expressed on a mass **(A)** and area **(B)** basis for *Lolium perenne* DGAT + CO5 (open circles; 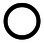) and NT control NT3 (closed circles; 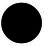) grown under 1–7.5 mM NO_3_^−^ supply. Photosynthesis measurements were made at 1500 μmol photons m^−2^ s^−1^.

Photosynthesis and leaf N correlated positively for NT3 and DGAT + CO5, regardless of whether measurements were expressed on a mass or area basis (Figure 7). The slope of the relationship between A_mass_ and leaf N_mass_ was, however, steeper for DGAT + CO5 than for NT3 across much of the leaf N_mass_ range observed (Genotype × N_mass_ interaction, *p* < 0.05; Figure 7). A_mass_ exhibited a saturating response to high leaf N_mass_ for DGAT + CO5 (Quadratic N_mass_ effect, *p* < 0.01; Figure 7). A_area_ (per unit leaf area) exhibited an even stronger saturating response to N_area_ beyond approximately 1.25 gN.m^−2^ for both DGAT + CO 5 and NT3 (Quadratic N_area_ effect, *p* < 0.001; Figure 7).

### Experiment 3: DGAT + CO5 Within-Leaf N Allocation and Rates of CO_2_ Diffusion

**Figure 8:**
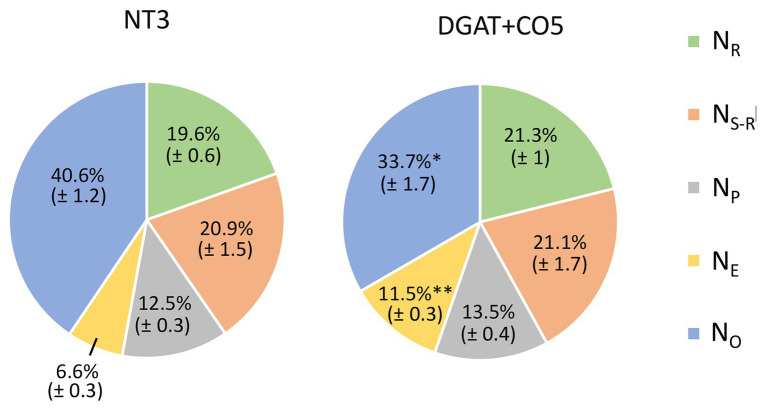
Within-leaf N partitioning for *L. perenne* DGAT + CO5 and NT control NT3 grown under 5 mM NO_3_^−^ supply. N_R_ (N invested in rubisco), N_S-R_ (N invested in non-rubisco soluble protein), N_P_ (N invested in pigment protein complexes), N_E_ (N invested in “bioenergetics”) and N_O_ (“other” N) as a proportion of total leaf N. Means ± SE, ^*^p < 0.05, ^**^p < 0.01, *n* = 6–8.

DGAT + CO5 displayed a significant decrease in total soluble protein and chlorophyll per unit leaf area, compared to NT3 (Table 3). In contrast, rubisco per unit leaf area did not significantly differ for DGAT + CO5 and NT3 (Table 3). DGAT + CO5 and NT3 invested similar proportions of leaf N into rubisco (N_R_/N) and pigment-protein complexes (N_P_/N; Figure 8), while DGAT + CO5 invested a significantly greater proportion of leaf N to bioenergetics (N_E_/N) than NT3 (Figure 8). Due primarily to this increase in N_E_/N, investment in “photosynthetic-N” × [(N_R_ + N_P_ + N_E_)/N] was significantly greater for DGAT + CO5 than for NT3 (Figure 8). The proportion of N invested in non-rubisco soluble protein (N_S_-N_R_)/N did not differ for DGAT + CO5 and NT3 (Figure 8) while investment in all remaining pools (N_O_/N) was significantly lower for DGAT + CO5 than for NT3 (Figure 8).

**Table 3:** Biochemical and gas exchange parameters for *Lolium perenne* DGAT + CO5 and NT control 3 weeks after defoliation.

Parameter		NT3	DGAT + CO5
N_mass_	%DW	3.2 (± 0.09)	4.1 (± 0.17)^**^
N_area_	gN m^−2^	1.9 (± 0.05)	1.5 (± 0.05)^**^
PNUE_sat_	μmol CO_2_ gN s^−1^	8.6 (± 0.32)	15.7 (± 0.37)^**^
Soluble protein	g m^−2^	4.8 (± 0.13)	3.96 (± 0.15)^**^
Rubisco	g m^−2^	2.3 (± 0.11)	2.02 (± 0.15)
Chl_A+B_	μmol m^−2^	453 (± 13)	386 (± 9)^**^
Chl_A:B_	mol mol^−1^	3.4 (± 0.01)	3.8 (± 0.02)^**^
Cyt*f*	μmol m^−2^	0.86 (± 0.06)	1.2 (± 0.02)^**^
α	mol mol^−1^ PAR	0.85 (± 0.001)	0.82 (± 0.001)^**^
α/Chl_A+B_	mol μmol^−1^	1.9 (± 0.01)	2.2 (± 0.04)^**^
CE	Dimensionless	0.08 (± 0.005)	0.1 (± 0.005)^**^
V_cmax_	μmol m^−2^ s^−1^	54 (± 1.4)	57.5 (± 1.1)
J_max_	μmol m^−2^ s^−1^	123.5 (± 9.6)	171.9 (± 2.9)^**^
R_d_	μmol m^−2^ s^−1^	0.71 (± 0.05)	0.81 (± 0.01)
C_i_*	μmol mol^−1^	28.5 (± 1.3)	26.8 (± 1.4)
Γ*	μmol mol^−1^	31.3 (± 1.5)	28.9 (± 1.5)
g_m_	mol m^−2^ s^−1^	0.29 (± 0.04)	0.4 (± 0.02)^*^
C_C_	μmol mol^−1^	226 (± 4)	237 (± 3)^*^
C_I_-C_C_	μmol mol^−1^	59.9 (± 5.8)	45.3 (± 2.6)^*^

N_mass_, leaf N concentration; N_area_, N per unit leaf area; PNUE_sat_; photosynthetic nitrogen use efficiency at 1500 μmol photons m^-2^ s^-1^; soluble protein; rubisco concentration; Chl_A+B_, total chlorophylls; Chl_A:B_, the ratio of chlorophyll *a* to chlorophyll *b*; Cyt*f*, cytochrome *f* contents; α, leaf absorptance; α/Chl_A+B_, leaf absorptance per unit chlorophyll; CE, carboxylation efficiency as determined by the initial slope of A-C_i_ regression; V_cmax_, maximum rubisco carboxylation rate; J_max_, maximum electron transport rate; R_d_, day respiration, C_i_*, intercellular CO_2_ compensation point; Γ*, chloroplastic CO_2_ compensation point; g_m_, mesophyll conductance; C_C_, chloroplastic CO_2_ concentration; C_I_-C_C_, CO_2_ drawdown. Means ± SE. *n* = 6–10.

* *p* < 0.05;

** *p* < 0.01.

There was no significant difference in intercellular CO_2_ concentrations (C_i_) measured between DGAT + CO5 and NT3. However, DGAT + CO5 exhibited a 39% increase in mesophyll conductance, compared to NT3 (Table 3). As such, chloroplastic CO_2_ concentrations (C_c_) were 5% greater, and the CO_2_ drawdown from substomatal cavities to chloroplasts (C_i_-C_c_) was 24% lower for DGAT + CO5, compared to NT3 (Table 3). The carboxylation efficiency (CE; initial slope of A-C_i_ response), the quantum efficiency of PSII (*φ*PSII) and J_max_ were all significantly greater for DGAT + CO5, whereas Ci*, Γ*, and V_cmax_, did not significantly differ between DGAT + CO5 and NT3 (Table 3).

## Discussion

In this study we described three experiments examining the relationships between photosynthesis, leaf N, and an engineered carbon sink in leaves of *L. perenne*. In experiment 1, we tested whether increasing sink capacity *via* DGAT + CO accumulation corresponded to increased photosynthesis. Across five independently transformed lines, the level of DGAT accumulation positively correlated with the relative increase in FA, which ranged from 118 to 174% of respective controls (Figure 1). For those lines with the largest increases in FA, DGAT + CO3-5, this carbon sink occurred at the expense of leaf sugar (Figure 2), and coincided with leaf-level changes that increased carbon assimilation, i.e., increased photosynthesis (DGAT + CO3-5) and SLA (DGAT + CO5). In experiment 2, we examined DGAT + CO5 photosynthesis under five levels of N availability. We found increased A_area_ for DGAT + CO5 compared to NT3 only occurred when NO_3_^−^ supply exceeded 3 mM (Figure 6) and that DGAT + CO expression made photosynthesis more responsive to variation in leaf N (Figure 7). For DGAT + CO3-5, N_mass_ was significantly higher compared to respective NT controls (Figure 4); however N on a per leaf area basis (N_area_) was either comparable to, or significantly lower than, respective NT controls (DGAT + CO3,4 and DGAT + CO5 respectively; Figure 4). Consequently, PNUE was higher for DGAT + CO3-5 compared to controls (Figure 4). To identify the physiological mechanism by which DGAT + CO delivers increased photosynthesis and PNUE, in experiment 3 we examined rubisco contents, within-leaf N allocation and g_m_ for DGAT + CO5 and NT3. We found no difference in rubisco, N allocated to rubisco or V_cmax_ between DGAT + CO5 and NT3 (Figure 8; Table 3). In contrast, DGAT + CO5 displayed significantly greater J_max_, N allocated to photosynthetic pools, and both stomatal (g_s_) and mesophyll (g_m_) conductance. Collectively, our data showed the addition of a novel carbon sink in leaves of *L. perenne*, at the expense of leaf WSC, can induce leaf level changes (e.g., increased g_m_ and N allocated to photosynthetic electron transport) which both increase A_area_ and PNUE. Moreover, we believe that this study is the first to estimate changes in N allocation resulting from manipulation of sink capacity.

### How Does DGAT + CO Expression Increase A_area_ and PNUE?

Regulation of photosynthetic capacity is determined by, among other things, the availability of carbon (source strength) relative to the demand for carbon (sink strength; (Arp, 1991; Paul and Foyer, 2001; Ainsworth et al., 2004) and sugar plays a key role in signaling carbon availability (Paul and Driscoll, 1997; Roitsch, 1999; Iglesias et al., 2002; Ainsworth and Bush, 2011; Ribeiro et al., 2017). Beechey-Gradwell et al. (2020) postulated that DGAT + CO lipid sinks may elevate the demand for carbon which could induce physiological and morphological changes which promote carbon capture (i.e., increased photosynthesis and SLA). Consistent with this hypothesis, we identified a significant negative correlation between WSC and photosynthesis in *L. perenne* leaves (Figure 5) and only those three lines with the largest FA increases and a significant reduction in leaf WSC (DGAT + CO3-5; Figure 2) displayed an increase in net photosynthesis and PNUE (Figure 3). Below, we discuss those leaf-level physiological changes which delivered increased photosynthesis and PNUE following DGAT + CO lipid sink accumulation.

A_area_ and PNUE are determined by a range of factors including the amount of light absorbed, the rate of CO_2_ transfer from the atmosphere to carboxylation sites, the proportion of N invested in photosynthesis, the fraction of photosynthetic-N devoted to the most rate-limiting photosynthetic processes, the specific activity and activation state of rubisco, and differences in respiration in the light (Poorter and Evans, 1998). Photosynthetic rate under growth PAR and CO_2_ conditions (600 μmol photons m-^2^ s^−1^ and 415 ppm CO_2_, respectively) appeared at the intersection of “rubisco-limited” and “RuBP-limited” in *L. perenne* (Supplementary Figure S1). Interestingly, neither rubisco content per unit leaf area nor N_R_/N significantly differed between NT3 and DGAT + CO5 (Table 3) and given the identical genetic backgrounds of these lines, rubisco likely had identical kinetic properties. However, both stomatal (g_s_) and mesophyll (g_m_) conductance were higher for DGAT + CO5 compared to NT3 (Tables 2, 3), collectively delivering a 5% increase in C_c_ at ambient CO_2_ (Table 3). Moreover, when g_m_ values were fixed in an A-C_i_ model (Sharkey et al., 2007), NT3 and DGAT + CO5 exhibited no significant difference in V_cmax_ (Table 3), suggesting enhanced g_m_ could account for the higher DGAT + CO5 carboxylation efficiency (CE; Table 3). Changes in g_m_ following sink capacity manipulation have previously been reported for rice (Detmann et al., 2012) and various legumes (Sugiura et al., 2018, 2020). Increased g_m_ may explain the reduced rubisco oxygenation to carboxylation ratio (V_o_/V_c_) previously reported for DGAT + CO *L. perenne* (Beechey-Gradwell et al., 2020) and contribute to the enhanced A_area_ and PNUE identified here.

RuBP-regeneration limited photosynthetic rate is typically attributed to insufficient electron transport (*J*). This can be alleviated by reducing photorespiration and its associated ATP costs, possibly achieved *via* increased g_m_. Alternatively, RuBP-regeneration limited photosynthesis could be enhanced with increases in the enzyme complexes that perform photosynthetic electron transport. In this study, thylakoid membrane-associated N was divided into two components, light harvesting (N_P_) and electron transport plus ATP synthesis (collectively “bioenergetics”; N_E_). We estimated cyt *f* and N_E_ indirectly, assuming that NT3 and DGAT + CO5 shared the same fixed relationship between J_max_, cyt *f* and N_E_ (Evans and Clarke, 2019). Under this assumption, DGAT + CO5 exhibited 73% higher N_E_/N than did NT3 (Figure 8), which could account for most (64%) of the difference in total photosynthetic N (N_R_ + N_P_ + N_E_) between the genotypes. However, available estimates of the N cost of bioenergetics vary and are highly sensitive to the amount of ATP synthase assumed (Evans and Clarke, 2019). For this reason, we additionally calculated the N_E_/N difference for DGAT + CO5 and NT3 by substituting an older, more conservative N_E_ cost of 8.85 mol N mmol^−1^ cyt *f* (Evans and Seemann, 1989) for DGAT + CO5 (c.f. 10.86 mol N mmol^−1^ cyt f for NT3). This did not alter the conclusion that DGAT + CO5 had a higher N_E_/N than did NT3 (46%; *p* < 0.001).

Changes in chlorophyll content also present an opportunity to improve PNUE (Slattery et al., 2017). Crop plants “overinvest” in N_P_ under high light (Evans and Poorter, 2001), and one proposed strategy to engineer higher A_area_ is to reduce chlorophyll in order to “free up” N for more rate-limiting processes (Slattery et al., 2017). In experiment 3, N_P_/N did not significantly differ for DGAT + CO5 and NT3 (Figure 8); however, DGAT + CO5 exhibited 15% lower Chl_A+B_ per unit leaf area than did NT3 (Table 3). Lower Chl_A+B_ penalizes light absorption which can reduce A_area_ at low irradiance but has less effect near saturating irradiance. Additionally, increases in light absorption (*α*) per unit of additional Chl_A+B_ diminish as Chl_A+B_ approaches 400 μmol m^−2^ (Evans and Poorter, 2001), values similar to that reported here (Table 3). For this reason, estimated absorptance (*α*) was only 2% lower for DGAT + CO5 than NT3, while estimated absorptance per chlorophyll molecule (α/Chl_A+B_) was 14% higher. Assuming the same pigment-protein stoichiometry for NT3 and DGAT + CO5 leaves (37.3 mol N mol^−1^ Chl_A+B_, as in Evans and Clarke, 2019), spreading chlorophyll over a greater leaf area would be expected to reduce the N cost of light harvesting and increase PNUE. However, pigment-protein stoichiometry and, therefore, the N cost of light harvesting vary naturally. For example, an increase in Chl_A:B_ during acclimation to high irradiance slightly increases the protein cost (and therefore N) of complexing pigments (Leong and Anderson, 1984; Evans and Seemann, 1989). DGAT + CO5 also exhibited a 10% higher Chl_A:B_ than NT3 (Figures 5, 6), perhaps indicative of a higher N cost of light harvesting which could partially offset the positive ΔPNUE due to higher α/Chl_A+B_.

### Other Factors Which Influence the DGAT + CO Photosynthetic Response

In experiment 1, only the three lines transformed from cultivar “Impact” (DGAT + CO3-5) displayed a reduction in leaf WSC content and an increase in photosynthesis and growth, whereas the two “Alto” cultivar transformed lines (DGAT + CO1-2) did not. The reason for this was unclear; either carbon allocation into lipids was too low in DGAT + CO1-2, or the response to DGAT + CO differs depending on the *L. perenne* cultivar used for transformation. It is worth noting that compared to either “Impact” control line (NT2-3), the “Alto” conrol line (NT1) displayed a lower leaf WSC content and a greater photosynthesis and growth rate. It may be that carbon utilization (e.g., translocation) was already high in the “Alto” background and there was little remaining capacity to enhance photosynthesis or growth *via* a new sink. This highlights an important consideration regarding the utility of engineered carbon sinks to improve photosynthesis. Benefits are likely to depend upon factors which influence the overall balance of activity between source leaves and various sinks throughout the plant, and thus may depend on environmental conditions, species, cultivar, or developmental stage. Crop species vary in both the capacity to accumulate carbohydrates in leaf cellular compartments (Chu et al., 2020) and in the sensitivity of photosynthesis to feedback regulation by carbohydrates (Sugiura et al., 2018). Thus, assessment of DGAT + CO photosynthesis in a range of plant backgrounds is needed to understand the broader applicability of our findings.

It is well-established that major carbon sinks in the form of reproductive structures and storage organs can influence the photosynthetic traits of source leaves (Ainsworth et al., 2004; Sugiura et al., 2015), but mature leaves themselves consist of various metabolic and structural sinks which compete for carbon (Vanhercke et al., 2019b). Manipulating leaf sink capacity though metabolic engineering may enhance photosynthesis if carbon-rich compounds can accumulate without triggering evolved carbon-sensing mechanisms (Paul and Eastmond, 2020). Encapsulated TAG appears to be capable of such an effect and, providing this energy-dense sink does not create excessive competition for carbon (Mitchell et al., 2020), an increase in net assimilation can be achieved (Beechey-Gradwell et al., 2020). Could other soluble and polymeric compounds (sugar derivatives, polysaccharides, proteins, or entirely novel bio-products such as vitamins, drugs, or plastics) beengineered to circumvent feedback inhibition? Creating an efficient carbon sink in metabolically active leaves is complex (Sweetlove et al., 2017). Introduced pathways must interfere minimally with desirable endogenous processes, and end-products should be metabolically inert or compartmentalized appropriately (Morandini, 2013). Futile cycles of synthesis and hydrolysis should be avoided (Winichayakul et al., 2013), and synthesis would ideally be turned on late in development when adequate source capacity has been established (Morandini, 2013). Despite this complexity, a growing range of options exists for fine-tuning the spatial and temporal synthesis of novel molecules in photosynthetic organisms (Sweetlove et al., 2017). A range of strategies by which leaf sink capacity might be enhanced remain to be explored, which could help to maintain photo-assimilate utilization and therefore maximize the photosynthetic potential of future crops.

## Data Availability Statement

The raw data supporting the conclusions of this article will be made available by the authors, without undue reservation.

## Author Contributions

LC, ZB-G, RS, GB, and NR designed the experiment. NR designed the DGAT + CO construct. KR transformed the material used in this study. LC and ZB-G conducted the three experiments and photosynthetic gas exchange, and wrote the paper. ZB-G conducted the Rubisco quantification and leaf N allocation. SW conducted the SDS-page analysis. PA conducted sugar and chlorophyll quantification. TC conducted FAMEs analysis. All authors contributed to the article and approved the submitted version.

### Conflict of Interest

The DGAT+CO ryegrass material examined in this study was generated with funding from DairyNZ, PGG Wrightson Seeds and Grasslanz Technology. The research conducted here, including all experimental designs and analyses was conducted in the absence of any commercial or financial relationships that could be construed as a potential conflict of interest.

## Footnotes

^1^http://www.gimp.org
