# Supplementary material for: Changes in Leaf-Level Nitrogen Partitioning and Mesophyll Conductance Deliver Increased Photosynthesis for Lolium perenne Leaves Engineered to Accumulate Lipid Carbon Sinks
Source: Front Plant Sci. 2021 Mar 9;12:641822. doi: 10.3389/fpls.2021.641822 (PMC8063613; doi:10.3389/fpls.2021.641822)
Supplement: Supplementary file 1 [file Data_Sheet_1.pdf]

Supplementary Material

Article title: Changes in leaf-level nitrogen partitioning and mesophyll conductance deliver increased photosynthesis for *Lolium perenne* leaves engineered to accumulate lipid carbon sinks.

Authors: Luke J. Cooney, Zac Beechey-Gradwell, Somrutai Winichayakul, Kim A. Richardson, Tracey Crowther, Philip Anderson, Richard W. Scott, Gregory Bryan, Nicholas J. Roberts.

1 Supplementary Tables

Supplementary Table S1. Experiment 1 mean (±SE) DW of five DGAT+CO lines and three non-transformed control lines at the time of propagation and 'post-establishment' i.e. three weeks after propagation. * or ** indicates statistically significant difference from respective non-transformed control ($P < 0.05$ and 0.01 respectively). n = 10. Lines with matching genetic backgrounds are grouped together.

	**Propagation DW (mg)**	**Post-Establishment DW (g)**
**WT1**	92.8 (± 5.1)	0.82 (± 0.04)
**DGAT+CO1**	101.7 (± 5)	0.7 * (± 0.02)
**DGAT+CO2**	101.8 (± 4.7)	0.82 (± 0.04)
**WT2**	107 (± 7.7)	0.57 (± 0.03)
**DGAT+CO3**	114.3 (± 5.5)	0.73 ** (± 0.03)
**DGAT+CO4**	105.6 (± 5)	0.72 * (± 0.05)
**WT3**	114.9 (± 10.6)	0.55 (± 0.02)
**DGAT+CO5**	94.1 (± 5.1)	0.65 (± 0.05)

Supplementary Table S2. Recombinant protein contents of DGAT and CO for five DGAT+CO lines.

	**Volume Intensity Unit**	
	**DGAT**	**CO**
**DGAT+CO1**	16538730	20278197
**DGAT+CO2**	8600768	12144132
**DGAT+CO3**	26666122	15308559
**DGAT+CO4**	24177690	26391414
**DGAT+CO5**	28964792	28302267

2 Supplementary Figures

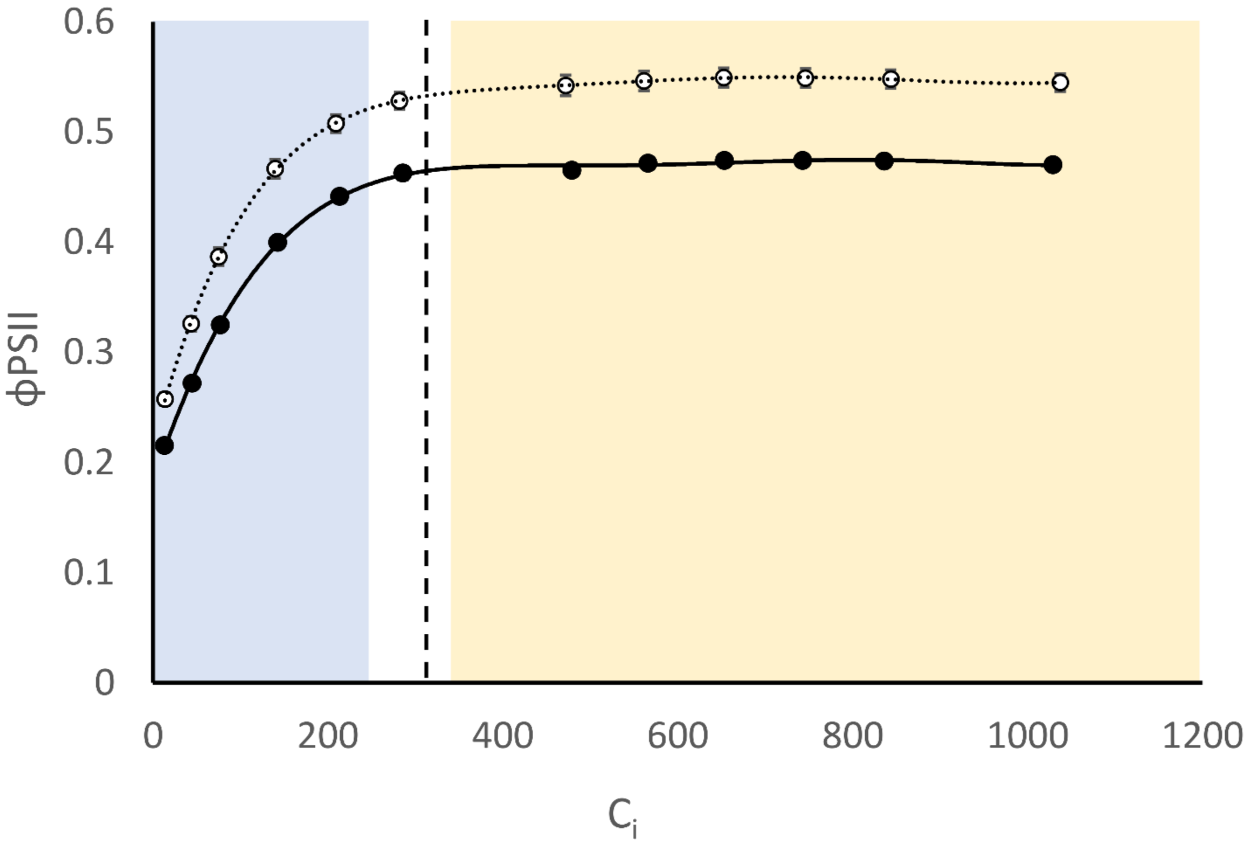

Supplementary Fig. S1. Quantum efficiency of PSII (ϕPSII) plotted against intercellular CO_2 (Ci) for *Lolium perenne* DGAT+CO5 (open circles ○) and non-transformed controls (NT3; closed circles ●) at 600 μmol photons m^{-2} s^{-1}. Rubisco-limited photosynthesis assigned between 0-250 ppm CO_2 (region shaded in blue), RuBP-regeneration limited photosynthesis assigned above 350 ppm CO_2 (region shaded in yellow). TPU limitation not evident. The region for which rate limitation was ambiguous is shaded white. Vertical dashed line represents C_i for both DGAT+CO5 and NT3 at ambient CO_2 concentrations (415 ppm CO_2). Means (±SE), n = 10.
